# Rituximab-Related Reversible Hepatocellular Damage

**DOI:** 10.5505/tjh.2012.98853

**Published:** 2012-12-05

**Authors:** Selami K. Toprak, Sema Karakuş

**Affiliations:** 1 Başkent University, School of Medicine, Department of Hematology, Ankara, Turkey

**To the Editor,**

Since its approval in 1997 by the US Food and Drug Administration, use of rituximab (MabThera^®^, Roche, Switzerland) has become widespread, especially for the treatment of non-Hodgkin lymphoma (NHL) and chronic lymphocytic leukemia (CLL) [1]. Rituximab is a monoclonal chimeric antibody that targets the CD20 antigen on the surface of normal B-cells and malignant cells in patients with CD20 B-cell lymphoproliferative disorders. The toxic effects of rituximab are mild and usually limited to its initial administration [2]. Reactions to its infusion include hypotension, fever, chills, rigors, urticaria, bronchospasm, angioedema, nausea, fatigue, headache, pruritus, dyspnea, rhinitis, vomiting, flushing, and pain at the disease site [3]. Such reactions usually occur at the beginning of the initial infusion within 30 min-2 h. Other possible and more serious adverse reactions are tumor lysis syndrome, mucocutaneous reaction, progressive multifocal leukoencephalopathy, hepatitis B reactivation with fulminant hepatitis, infection, cardiac arrhythmias, renal toxicity, and bowel obstruction and perforation [3,4]. A moderate increase in liver function test findings—depending on monoclonal anti-CD20 treatment—have been reported; however, no cases of a 15-20-fold increase in transaminases have been reported. Herein we present a case of rituximab treatmentrelated deterioration in liver function test results in a patient with CLL.

## CASE REPORT

A 50-year-old female was given 2 cycles of fludarabine/ cyclophosphamide (FC) combination therapy as a first line treatment for Rai stage II CLL. She was then given 2 cycles of rituximab/fludarabine/cyclophosphamide (R-FC) treatment, and was admitted to our clinic for preparation and evaluation before her fifth cycle of treatment. Her workup was within normal limits and, as such, on d 1 she was given rituximab 375 mg/m^2^ (total dose: 600 mg/d) during monitorization and for a time period in accordance with its prospectus information. Although the results of all tests performed before administration of the drug were normal, the patient’s alanine aminotransferase (ALT), aspartate aminotransferase (AST), lactate dehydrogenase (LDH), alkaline phosphatase (ALP), and gamma glutamyl transferase (GGT) levels increased sharply the day following administration (Table). The patient had no clinical complaints and her physical examination did not show any associated pathology. In addition, the D-dimer level, prothrombin time, bilirubin level, and hemolysis test results were within normal limits, as were blood electrolytes, kidney function test results, and the whole blood count. 

The patient’s history of other medical conditions was negative and she did not report regular use of any medication. Administration of the scheduled subsequent chemotherapeutics (FC) according to the treatment protocol was delayed. Hepatobiliary ultrasonography was performed and the results were normal. Screening for hepatitis A, B, and C, and other infectious serologies, including Epstein- Barr virus, cytomegalovirus, human immunodeficiency virus, toxoplasma gondii, rubella, herpes zoster, and herpes simplex, was performed, all of which were negative for acute infection. Autoimmune serologies, including antiliver kidney microsome, anti-smooth muscle antibody, and anti-nuclear antibody, were also negative. The patient was referred to the gastroenterology department for consulta-tion and was followed-up with daily liver function testing and intravenous hydration. She required no additional treatment and on d 7 of the above-described treatment, as her laboratory parameters regressed to basal values, the FC combination therapy was successfully completed. No clinical or laboratory problems were encountered and the patient was discharged with scheduled close-monitoring follow-up.

## DISCUSSION

Based on the presented patient’s negative viral and autoimmune serologies, and rapid recovery after cessation of rituximab therapy, and the fact that rituximab was the only drug the patient received immediately preceding liver function deterioration, we think that this was a case of monoclonal anti-CD20 drug-induced impairment. The half-life of rituximab is approximately 22 d and it is detectable months after administration; however, the presented patient developed abnormal liver function 1 d after administration of the drug, when the concentration is expected to be high [5]. 

Several viral infections associated with rituximab, particularly viral hepatitis, have been reported [6]. Viral reactivation is a well-known side effect of the drug; however, the presented case highlights the possibility that direct hepatotoxicity may result from rituximab therapy. The question that remains is why rituximab caused no side effects the first 2 times it was given to this patient, but did result in toxicity when administered the third time. Winkler et al. studied 11 patients that underwent rituximab treatment for fludarabine-resistant recurrent CLL/ NHL and reported that serum concentrations of liver enzymes, including ALT, AST, and GGT, increased to levels that exceeded the normal range by a factor greater than 5, whereas the concentration of ALP, and direct and indirect bilirubin remained stable throughout antibody treatment [7]. Additionally, the LDH level increased in 9 of the patients during treatment, peaking in 2 of the patients at values >2,000 U/mL [7]. 

In the presented patient the marked increase in peripheral lymphocytes was associated with elevated LDH and liver transaminases; however, the lymphocyte count remained in the normal range and not differ from previously obtained values. Researchers have suggested that in order to prevent rituximab-related toxicity it is reasonable to lower the number of circulating tumor cells to <50.0 x 10^9^/L using chemotherapeutic regimens prior to administering rituximab. In contrast to our expectations based on the literature, in the presented case limited and reversible hepatocellular damage due to rituximab was thought to occur directly, instead of triggering viral reactivation, and presented with laboratory findings only—there were no associated clinical signs. As such, we recommend close follow-up of transaminases following rituximab treatment. 

**Ethical Consideration **

Written informed consent was obtained from the patient. 

**Conflict of Interest Statement**

None of the authors have any conflicts of interest, including specific financial interests, relationships, and/or affiliations, relevant to the subject matter or materials included.

## Figures and Tables

**Table 1:**
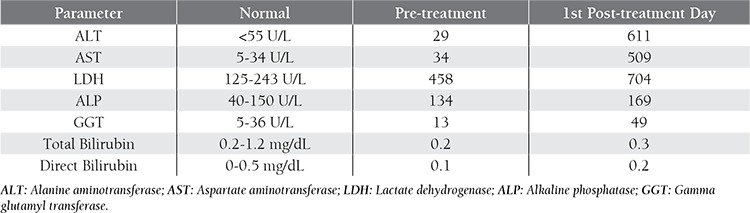
Liver function biomarker levels.
